# Chemical fractions of potassium in arid region calcareous soils: The impact of microclimates and physiographic variability

**DOI:** 10.1371/journal.pone.0314239

**Published:** 2024-11-25

**Authors:** Morteza Poozesh Shirazi, Sayyed Mahmoud Enjavinezhad, Ali Akbar Moosavi

**Affiliations:** 1 Department of Soil and Water Research, Fars Agricultural and Natural Resources, Research and Education Center, AREEO, Shiraz, IR Iran; 2 Department of Soil Sciences, College of Agriculture, Shiraz University, Shiraz, IR Iran; University of Ferrara: Universita degli Studi di Ferrara, ITALY

## Abstract

Factors such as topography, mineralogy, physicochemical properties, and climate can affect the distribution of soil potassium (K) forms. However, often the special effects of physiographic units are neglected. Therefore, this study aimed to investigate the factors controlling the distribution of chemical fractions of K in different physiographic units of calcareous soils (piedmont plain, flood plain, alluvial plain, lowland, badland, and plateau) in southern Iran. The XRD analyzing patterns showed that the distribution of K forms is controlled by K-bearing minerals (i.e., illite) in finer particles of the soils. Physiographic units significantly affect the distribution of K forms due to different microclimates (such as soluble, exchangeable, and non-exchangeable). In addition, different correlations between the K forms and some physicochemical properties of the soils such as soil texture (i.e., silt (r = 0.511** to 0.527**) and sand (r = -0.357* to -0.389*)), electrical conductivity (EC) (r = 0.617** to 0.723**), sodium absorption ratio (SAR) (r = 0.478** to 0.577**), pH (r = 0.347* to 0.519**), and gypsum (r = 0.372* to 0.475**) were found in soils of the study area. It is recommended that cultivation sites are chosen with a deeper understanding of land conditions e.g., slope, elevation, microclimatic conditions, soil development, and clay mineralogy.

## Introduction

Potassium (K) is considered an essential element in plant nutrition. Several roles of this nutrient have been documented, such as seed germination, enzyme activation, protein synthesis, absorption and transport of ions, photosynthesis, respiration, productivity, essential oil components, and yield of plants [1–3]. In addition, enzymatic processes and ATP synthesis are also affected by K, which leads to extreme drought damage in plants that are suffering from K deficiencies. Furthermore, one key and beneficial role of K in plants is improving protection against frost injury and winter kill due to its high tolerance of low temperatures [4]. Various crop physiological processes that control quality, growth, chemical composition, and stress resistance are also done by the presence of K [5,6].

Comprising the Earth’s crust with an average of about 2.3%, this element turns out to be the seventh most abundant element and the fourth most abundant mineral nutrient in the lithosphere [7]. The most abundant K-bearing minerals presented in the soils are primary minerals such as feldspars and micas, and secondary minerals such as illite and transitional clays. Despite presenting high total K content in most soils, only a small portion is immediately and slowly accessible for plant uptake [8]. A long-term non-application of K and intensive cropping would cause a large amount of soil K to be depleted [9]. Certain regions of the world, such as Australia, China, and Iran, are suffering from K deficiency in crops on large scales due to specific climatic conditions or long-time fertilization [10]. Contrary to such observations, in Europe, soil K deficiency is not that widespread, but the reduction of this element and consequently its deficiency has been reported on a regional scale, especially in the Baltic countries and the United Kingdom [11].

Soil K is divided into four chemical forms according to its availability to the plants, including soluble K, exchangeable K, non-exchangeable K, and structural K [12]. Some important soil and environmental factors control the variation of K forms in different soil depths or areas [13]. These factors include soil parent material, soil weathering rate, topography, and nutrient exchange [14]. Among these four chemical K forms, soluble and exchangeable K forms are rapidly released into the soil solution and are available for plants. These forms of K are higher in the surface soil layer than in the subsurface soil layer [15]. Non-exchangeable K has a key role in supplying available K which is fixed in the interlayer of 2:1 clay minerals and could be released slowly [16]. Structural K comprised a major portion of total K content (nearly 98%) and its amount varied due to the composition of the parent rock and soil development process [17]. Factors such as mineralogy particularly mica, development, salinity, and some soil physicochemical properties could be involved in distributing soil K forms. In addition, one of the most important influential soil-forming factors affecting the distribution of K forms is climate which can affect calcareous soils by its two major parameters of temperature and precipitation [18]. Other factors such as calcium carbonate equivalent content, clay content, and cation exchange capacity are influenced by climate [19].

The important effect of topography, as one of the soil forming factors, on soil characteristics is well documented. This factor deals with the earth’s surface configuration which includes the overall shape of the ground and the height of positions [20]. Soil genesis in toposequences can be studied by some physicochemical experiments while topography is the most variable soil-forming factor [21]. Topography factors directly and indirectly affect soil physicochemical properties, including the percentage of clay, organic matter, soil reaction, calcium carbonate content, mineralogy, color, moisture content, and even the concentration of nutrients such as phosphorus and iron [22]. Slope position, soil depth, and mineralogy are documented as variables that control distribution of soil K forms [23]. The effect of topographic positions on soil properties showed high clay content and available nitrogen (N), phosphorous (P), and K in lowland positions compared to upland and midland [24].

The south of Iran is located in semi-arid and arid regions of the world. In these regions, topography has a great influence on soil development and characteristics. Effects of topography on distribution of soil K forms have been studied by some researchers [12,25,26]. Whereas, the effect of physiographic units on the distribution of K forms particularly in the toposequences of arid and semi-arid regions has been studied on rare occasions [14]. Therefore, the present study was conducted to investigate the distribution of K forms along different topographic units with special emphasis on the role of different physiographic units in Bushehr Province, Iran.

## Materials and methods

### Study area

**Fig 1:**
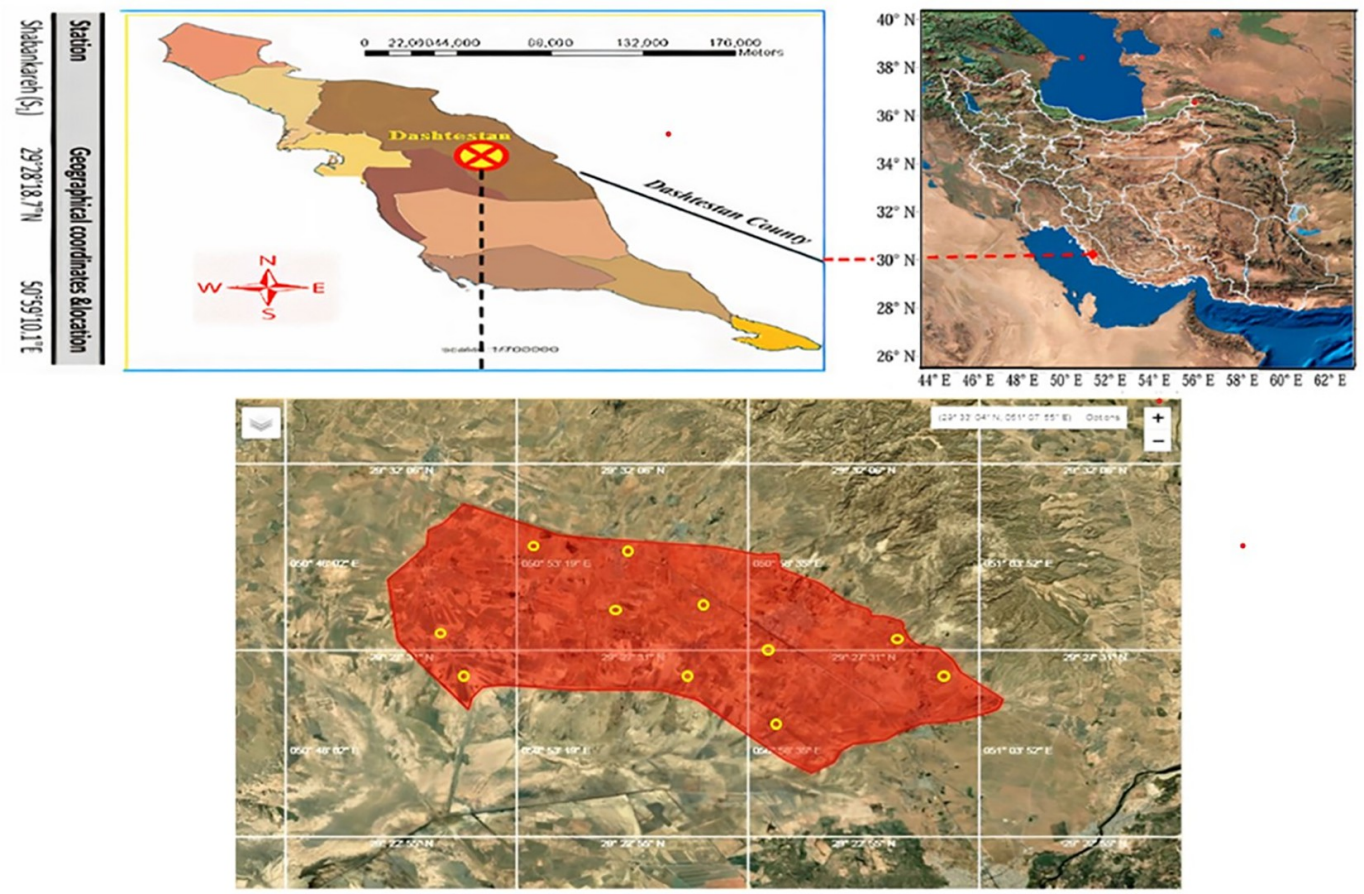
Study area along with the sampling locations (the original maps were extracted from https://earthexplorer.usgs.gov and some needed modifications were performed by the authors).

The present study was conducted in the downstream lands of Rais Ali Delvari dam in Shabankareh, Dashtestan region of Bushehr Province, southern Iran with an area of 15,000 ha (50° 04´ 18´´ to 51° 51´ 02´´ eastern longitude and 29° 24´ 30´´ to 29° 29´ 18´´ northern latitude) (Fig 1). Commonly cultivated crops in the study area are wheat (*Triticum spp*), barley (*Hordeum vulgare*), sesame (*Sesamum indicum*), canola (*Brassica napus*), and different types of vegetables. The study area’s soil temperature and moisture regimes are hyperthermic and aridic, respectively.

### Sampling and sample preparation

Different physiographic units were separated using Google Earth images topographic maps with a scale of 1:50000. 11 soil profiles were selected for sampling [27]. Profile description was performed according to the soil profile description guide [28]. Sufficient soil samples (about 2 kg) were taken from the identified horizons and after transferring to the laboratory, the samples were air-dried passed through a 2 mm sieve and kept for further physicochemical analysis.

### Soil physical and chemical analysis

Some basic soil physical and chemical attributes were determined using the following standard methods: soil pH and electrical conductivity (EC) by using a glass-electrode pH-meter and EC-meter in the saturated paste and its extract, respectively; soil particle size distribution (PSD) by the hydrometer method [29,30]; calcium carbonate equivalent (CCE) by HCl neutralization [31]; gypsum percentage using the acetone method [32]; organic carbon (OC) using the wet oxidation method [33]; cation exchange capacity (CEC) by substitution of exchangeable cations with sodium acetate and replacement of sodium with ammonium acetate [34]; and soluble ions of sodium (Na^+^), calcium (Ca^2+^), and magnesium (Mg^2+^) in the saturated extract by flame photometer [35]. Furthermore, the sodium absorption ratio (SAR) was calculated using Eq (1)

$$SAR=\frac{{Na}^{+}}{\sqrt{\frac{({Ca}^{2+}+{Mg}^{2+}}{2}}}$$ (1)

where Na^+^, Ca^2+^, and Mg^2+^ are soluble forms of the mentioned ions (meq L^-1^).

### Determining chemical forms of K

The K content presented in different forms was extracted using the summarized methods introduced by Helmke and Sparks [36] as follows. The K content of all extracts was determined by the Jenway PFP7 flame photometer.

### Soluble and exchangeable K

A 1:5 (soil: water) suspension was used to extract soluble K. To extract exchangeable K, 5 g of soil from each sample with 25 mL of ammonium acetate 1 N (pH = 7) was shaken 10 minutes four times, then centrifuged. The supernatants were then collected in a volumetric flask and were equaled to 100 mL by ammonium acetate 1N (pH = 7).

### Non-exchangeable K

To extract non-exchangeable K, boiling nitric acid treatment was used. 2.5 g of soil and 25 ml of 1 N nitric acid were boiled for 10 min and then the extract being filtered was brought to a volume of 100 mL and then non-exchangeable K was calculated by subtracting the amount of K extracted by nitric acid, from the K extracted from ammonium acetate.

### Total K

To extract the total content of soil K, 0.5 g of soil was added to 0.5 mL of aqua regia (a mixed solution of concentrated nitric acid and concentrated hydrochloric acid (1:3 ratio)) and 10 mL of hydrofluoric acid was heated for 3 hours at 110°C. The mixture was then transferred to a plastic volumetric containing 2.8 g of boric acid and was brought to a volume of 100 mL.

### Clay mineralogy

Four soil profiles were selected with different physiographic units for performing clay mineralogy experiments. For clay mineral sample preparation, the cementing agents of samples including carbonates, organic matter, and iron oxides were removed by 1 N HCl, 30% H_2_O_2_, and dithionite citrate bicarbonate mixed solution, respectively [37,38]. After the separation of clay fraction, samples were saturated with Mg^2+^ and K^+^ using 1 N MgCl_2_ and 1 N KCl, respectively. The K^+^- and Mg^2+^-saturated samples were heated at 550°C and were saturated by ethylene glycol. The treatments were analyzed using the X-ray diffraction method with an 1130 Philips X-ray Diffractometer. The relative abundance of clay minerals, based on peak intensities was semi-quantitatively measured by the method of Johns et al. [39].

### Statistical analysis

The SPSS23 software package was used to determine the degree of correlation between data. One-way ANOVA was used to compare the amount of different extracted K forms between different physiographic units. Duncan’s multiple range test was used to better understand the differences and validate their results. In the mean comparison, if the sig value provided by the software is less than 0.05 (i.e., the probability level), the assumption of the equality of the means is rejected, and the difference between the populations is evident [14].

Note: This study was conducted in the downstream lands of Rais Ali Delvari dam in Shabankareh, Dashtestan region of Bushehr Province, southern Iran which is one of the study areas studied by Soil and Water Research Department, Agricultural and Natural Resources Research Center, AREEO, IR Iran. It should be noted that the first author is academic staff of AREEO and no permission was required to access the field site.

## Results

### Soil classification of the study area

**Fig 2:**
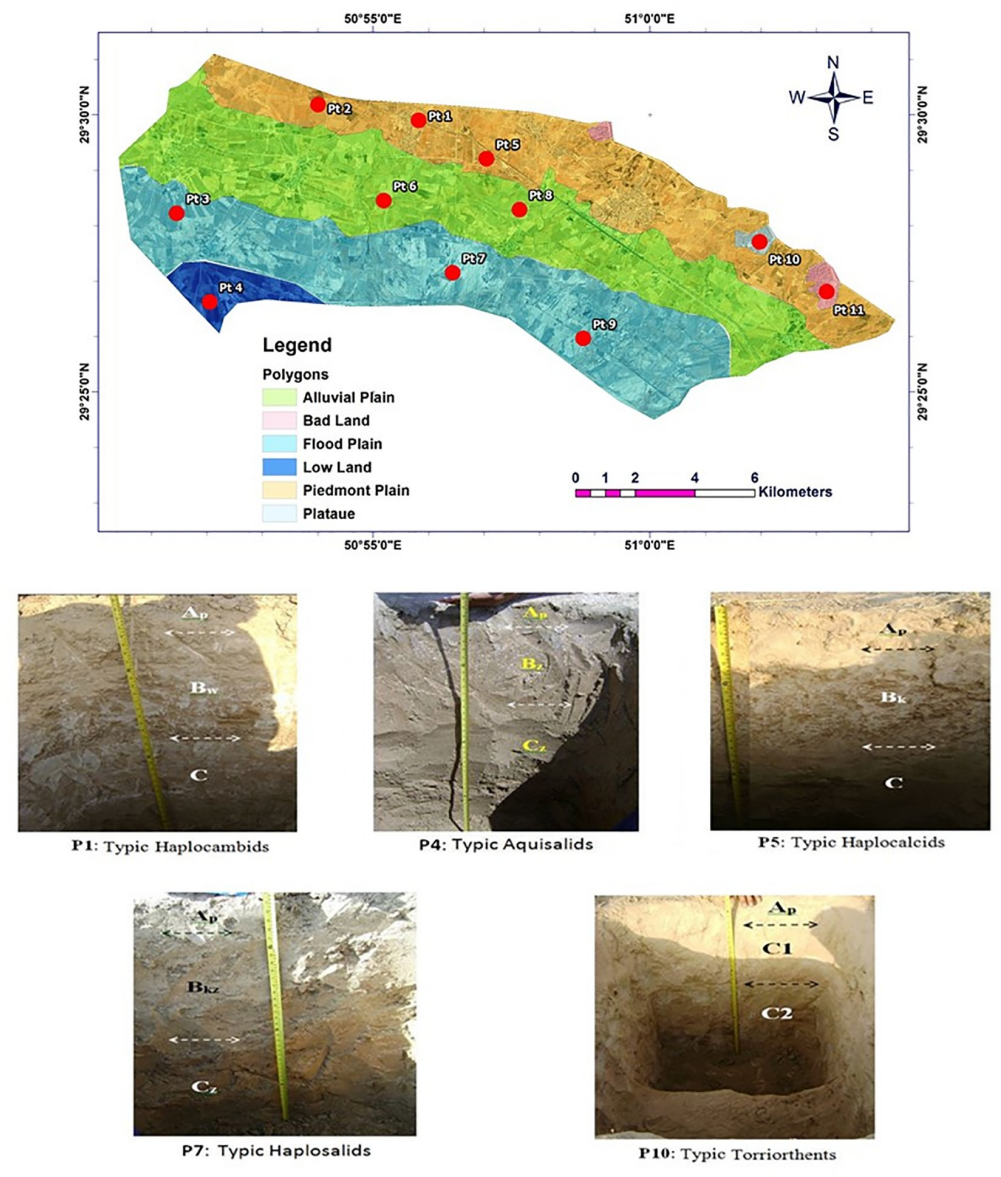
Identified physiographic units and some of the described soil profiles in the study area (the original maps were extracted from https://earthexplorer.usgs.gov and some needed modifications were performed by the authors).

The selected soil physicochemical properties and classification of the studied soil profiles are shown in Table 1. Based on Google Earth images and topographic maps with a scale of 1:50000, different identified physiographic units included piedmont plains, flood plains, alluvial plains, lowlands, badlands, and plateaus in the study area (Fig 2). Loam was the predominant soil texture in the study area. Soil pH was in the range of 7.50 to 7.80 with a mean value of 7.52, while the value of EC in the study area was in the range of 2.43 to 67.40 dS m^-1^ with a mean value of 17.64 dS m^-1^. The percentage of soil CCE was in the range of 23 to 65% with a mean value of 43.90%, while gypsum content was in the range of 1.50 to 5.50% with a mean value of 3.40%. Soil OC content was in the range of 0.10 to 0.28% with a mean value of 0.18%. The CEC of soils in the study area was 3.10 to 16.56 cmol_(+)_ kg^-1^ with a mean value of 8.10 cmol_(+)._kg^-1^; while SAR was in the range of 0.9 to 252.8 meq L^-1^ with a mean value of 31.73 meq L^-1^. Soils of the study area were classified into Aridisols (9 profiles) and Entisols (2 profiles) based on the Soil Taxonomy 2014 [28].

**Table 1:** Soil physicochemical properties of soil profiles along with their classifications based on the Soil Taxonomy 2014 [28].

Horizon	Depth (cm)	Textural class	Clay (%)	Silt (%)	Sand (%)	OC (%)	CCE (%)	CEC (cmol_(+)_ kg^-1^)	EC (dS m^-1^)	pH	Gypsum (%)	SAR ($\sqrt{meq\;L-1}$)
**Profile 1:** Typic Haplocambids **Physiographic unit:** Piedmont plain												
A	0–20	Sandy loam	4.20	32.00	63.80	0.23	26.50	3.60	2.43	7.20	2.00	0.92
Bw	20–50	Sandy loam	4.20	26.70	69.10	0.17	23.00	3.20	2.71	7.20	1.60	0.95
C	50–110	Sandy loam	4.20	32.70	63.10	0.10	30.50	3.10	2.57	7.20	1.80	0.90
**Profile 2:** Typic Haplocalcids **Physiographic unit:** Piedmont plain												
Ap	0–25	Silty clay loam	32.20	50.70	17.10	0.25	33.50	16.56	4.70	7.20	1.80	3.78
Bw	25–50	Loam	11.10	42.70	46.20	0.19	28.00	6.01	4.70	7.20	2.20	4.04
Bk	50–90	Loam	18.20	40.70	41.10	0.17	41.00	9.56	4.60	7.20	1.50	2.11
C	90–120	Loam	18.10	38.70	43.50	0.12	32.00	8.20	4.50	7.20	1.80	2.08
**Profile 3:** Typic Torriorthents **Physiographic unit:** Flood plain												
Ap	0–25	Loam	18.20	46.70	35.10	0.21	33.50	10.50	26.21	7.60	4.90	33.60
C	25–110	Loam	26.20	44.70	29.10	0.11	29.00	13.50	10.45	7.60	4.10	16.10
**Profile 4:** Typic Aqiusalids **Physiographic unit:** Low land												
Ap	0–10	Silt loam	13.50	56.70	29.80	0.21	50.00	7.21	67.40	7.80	3.20	252.80
Bz	10–35	Loam	20.50	49.30	30.20	0.15	49.50	10.60	53.20	7.80	3.60	78.80
Cz	35–110	Loam	21.50	48.70	29.80	0.12	51.50	11.21	38.10	7.80	4.00	74.10
**Profile 5:** Typic Haplocalcids **Physiographic unit:** Piedmont plain												
Ap	0–20	Loam	15.50	40.00	44.50	0.13	63.50	8.21	4.40	7.50	3.70	2.90
Bk	20–50	Sandy loam	7.50	40.00	52.50	0.13	65.00	5.80	6.70	7.50	4.20	8.70
C	50–110	Sandy loam	7.50	34.00	58.50	0.10	59.20	4.60	7.60	7.50	4.60	8.80
**Profile 6:** Typic Haplocalcids **Physiographic unit:** Alluvial plain												
A	0–20	Sandy loam	13.50	34.00	52.50	0.23	63.50	7.21	11.30	7.60	5.10	4.80
Bk	20–35	Loam	17.50	48.00	34.50	0.18	46.00	9.20	12.40	7.60	5.50	10.30
Bw	35–60	Silt loam	21.50	50.70	27.80	0.15	37.00	11.30	9.20	7.60	4.30	12.60
C	60–110	Silt loam	21.50	60.00	18.50	0.14	45.50	11.30	13.40	7.60	4.80	18.20
**Profile 7:** Typic Haplosalids **Physiographic unit:** Piedmont plain												
Ap	0–20	Silt loam	7.50	52.00	40.50	0.26	36.00	4.20	50.40	7.80	5.50	70.50
Bkz	20–70	Silt loam	17.50	52.30	30.20	0.18	47.00	9.21	35.30	7.80	5.20	77.30
Cz	70–110	Silt loam	8.30	54.80	36.90	0.12	42.50	6.20	32.00	7.70	5.40	67.50
**Profile 8:** Typic Haplocambids **Physiographic unit:** Alluvial plain												
Ap	0–15	Loam	30.20	45.60	34.20	0.28	54.50	15.50	17.30	7.60	3.30	21.20
Bw	15–50	Loam	22.20	49.60	28.20	0.23	50.00	11.50	5.80	7.60	2.60	7.30
C	50–110	Sandy loam	12.20	33.60	54.20	0.15	53.50	6.50	5.40	7.60	2.10	7.20
**Profile 9:** Calcic Haplosalids **Physiographic unit:** Flood plain												
Ap	0–20	Silt loam	8.20	67.60	24.20	0.20	38.00	4.56	55.30	7.70	5.30	93.60
Bkz	20–70	Silt loam	18.20	59.60	22.20	0.13	45.00	9.38	42.20	7.70	5.20	91.70
C	70–120	Silt loam	23.50	52.70	23.80	0.11	40.00	12.21	42.60	7.70	4.40	84.90
**Profile 10:** Typic Torriorthents **Physiographic unit:** Plateau												
Ap	0–15	Sandy loam	7.50	38.70	53.80	0.21	45.00	4.21	4.10	7.40	1.80	1.40
C1	15–50	Sandy loam	9.50	36.30	54.20	0.16	42.00	6.84	4.40	7.40	1.70	3.40
C2	50–110	Sandy loam	9.50	32.70	57.80	0.12	45.00	5.71	4.90	7.40	1.80	6.20
**Profile 11:** Typic Haplocambids **Physiographic unit:** Badland												
Ap	0–20	Sandy loam	9.50	32.70	57.80	0.27	50.00	6.21	4.20	7.50	1.60	1.70
Bw	20–45	Sandy loam	5.50	34.70	59.80	0.24	49.50	5.26	4.40	7.50	1.80	3.20
C	45–110	Loam	11.50	40.70	47.80	0.18	47.00	6.80	5.20	7.50	3.10	5.40

EC: Electric Conductivity of saturated extract; CCE: Calcium carbonate equivalent; CEC: Cation exchange capacity; OC: Organic carbon; SAR: Sodium absorption ratio.

### Clay mineralogy

Based on the difference in the type of physiographic units and soil profile development, four control soil profiles were selected to study clay minerals in different physiographic units. The relative abundance of clay fractions in the study area is presented in Table 2.

**Table 2:** Semi-quantitative analysis of clay content in the studied soils.

Soil profile	Horizon	Mixed layer	Chl	Ill	Kao	Mont
**4**	Ap	+	+++	++	++	+
	Cz	+	++	+++	++	+
**7**	Ap	+	+++	++	++	Tr
	Bkz	+	++	++	++	++
	Cz	+	++	+++	++	+
**8**	Ap	+	+	+++	++++	Tr
	Bw	+	++	++	+++	+
	C	+	++	+++	+++	Tr
**10**	Ap	Tr	++++	++	++	Tr
	C2	+	++	++	+++	+

Mont, montmorillonite; Ill, illite; Chl, chlorite; Kao, kaolinite; Tr, trace; +, content <15%; ++, content in the range of 15%–30%; +++, content in the range of 30%–50%; ++++, content >50%.

The mixed interlayer minerals, kaolinite, illite, and chlorite were the mainly observed minerals, while quartz and feldspars were also found in trace amounts in the studied soil profiles. These main minerals were observed in all soil surface and subsurface horizons. A similar trend was observed along with slight changes in the distribution of these minerals with increasing in depths of the horizons of all studied soil profiles.

### Distribution of K forms

The distribution of different K forms is shown in Table 3. The amount of soluble K form in the study area ranged from 4.29 to 44.07 mg kg^-1^ with a mean value of 13.72 mg kg^-1^; besides its relative percentage ranged from 0.10 to 0.83% with a mean value of 0.26% of the total K content. The minimum and maximum amounts of soluble K were found in the C horizon of profile no 8 and the Ap horizon of profile no 9, respectively. This form of K in the studied physiographic units varied in the following order: alluvial plain< plateau< piedmont plain< bad land< low land< flood plain.

**Table 3:** Distribution of different forms of K in the studied soils.

Soil potassium Forms (mg Kg^-1^)								Soil potassium Forms (%)			
Profile			Soluble potassium	Exchangeable potassium	Non-exchangeable potassium	Structural potassium	Total potassium	Soluble potassium	Exchangeable potassium	Non-exchangeable potassium	Structural potassium
**1**	**Minimum**		5.85	144.42	140.48	3828.06	4020.18	0.12	3.04	2.96	95.22
	**Maximum**		10.14	164.28	192.12	4643.94	4812.26	0.21	3.68	4.78	97.04
	**Mean**		7.67	152.24	166.97	4358.56	4525.53	0.17	3.38	3.75	96.25
**2**	**Minimum**		5.46	138.12	112.98	4135.17	4248.15	0.10	2.74	2.66	96.23
	**Maximum**		8.58	226.16	240.26	6831.66	7024.18	0.14	3.68	3.77	97.34
	**Mean**		6.73	178.24	178.48	5347.69	5526.17	0.12	3.27	3.23	96.77
**3**	**Minimum**		15.60	206.22	252.24	5206.16	5458.40	0.29	3.78	4.62	95.23
	**Maximum**		41.34	310.96	360.88	7210.18	7571.06	0.55	4.11	4.77	95.38
	**Mean**		28.47	258.59	306.56	6208.17	6514.73	0.42	3.94	4.69	95.31
**4**	**Minimum**		17.16	180.42	190.50	4722.14	5070.4	0.34	3.28	3.48	93.13
	**Maximum**		39.39	282.10	348.30	5486.61	5687.01	0.69	5.56	6.87	96.52
	**Mean**		26.26	216.30	246.40	5162.94	5409.34	0.48	4.05	4.63	95.37
**5**	**Minimum**		7.41	138.32	112.46	5257.75	5370.21	0.11	2.07	2.09	96.68
	**Maximum**		11.31	186.12	216.60	6783.83	6984.19	0.17	2.85	3.32	97.91
	**Mean**		8.97	156.24	176.47	6115.76	6292.23	0.14	2.50	2.76	97.24
**6**	**Minimum**		5.85	170.32	112.12	3953.51	4110.29	0.12	3.39	2.39	96.19
	**Maximum**		11.31	186.70	156.78	4910.08	5030.16	0.28	4.24	3.81	97.61
	**Mean**		7.70	177.87	132.47	4462.23	4594.70	0.17	3.89	2.92	97.08
**7**	**Minimum**		10.14	216.16	200.18	4795.88	5024.26	0.20	4.02	3.72	92.90
	**Maximum**		35.49	370.80	384.20	5176.11	5414.91	0.66	6.85	7.10	96.28
	**Mean**		18.98	274.40	270.92	5000.90	5271.82	0.36	5.19	5.12	94.88
**8**	**Minimum**		4.29	164.68	68.74	4163.37	4232.11	0.10	3.72	1.62	96.65
	**Maximum**		7.80	216.18	168.74	5051.73	5180.15	0.15	4.29	3.35	98.38
	**Mean**		5.98	191.21	121.97	4696.85	4818.82	0.12	3.97	2.48	97.52
**9**	**Minimum**		17.16	226.60	216.84	4745.60	4962.44	0.35	4.57	4.37	92.96
	**Maximum**		44.07	390.34	372.12	4920.44	5283.13	0.83	7.39	7.04	95.63
	**Mean**		28.47	312.41	288.38	4859.02	5147.40	0.55	6.04	5.58	94.42
**10**	**Minimum**		5.46	180.60	100.92	4179.23	4280.15	0.10	4.22	2.36	95.42
	**Maximum**		19.11	280.88	264.86	6109.63	6374.49	0.30	4.87	4.58	97.64
	**Mean**		10.14	239.23	202.09	5101.51	5303.59	0.18	4.50	3.70	96.30
**11**	**Minimum**		6.63	192.12	120.02	3392.15	3512.17	0.17	4.38	2.97	94.70
	**Maximum**		17.55	272	216.72	4585.77	4726.21	0.43	6.65	5.30	97.03
	**Mean**		10.79	223.69	159.06	3949.88	4108.94	0.26	5.50	3.90	96.10
**Study area**	**Minimum**		4.29	138.12	68.74	3193.4	3512.17	0.10	2.07	1.62	84.73
	**Maximum**		44.07	390.34	384.2	6857.88	7571.06	0.83	7.39	7.10	95.18
	**Mean**		13.72	212.91	198.64	4755.50	5180.76	0.26	4.17	3.81	91.75

The amount of exchangeable form of K in the study area ranged from 138.12 to 390.34 mg kg^-1^ with a mean value of 212.91 mg kg^-1^, and the relative percentage of this form of K varied from 2.07 to 7.39% with a mean value of 4.17% of the total K content. The minimum and maximum amounts of this form of K were found in the Bk horizon of profile no 2 and the Ap horizon of profile no 9, respectively. Regarding different physiographic units, the following trend for variation of the exchangeable form of K was found: alluvial plains < piedmont plains < lowlands < badlands < plateau < flood plains.

The non-exchangeable form of K ranged from 68.74 to 384.20 mg kg^-1^ with a mean value of 198.64 mg kg^-1^. The relative percentage of the non-exchangeable form of K ranged from 1.62 to 7.10% with a mean value of 3.81%. The minimum and maximum amounts of this form of K were found in the C horizon of profile no 8 and the Ap horizon of profile no 7, respectively. This form of K varied as the following trend in different physiographic units: alluvial plains < badlands < piedmont plains < plateau < lowlands < flood plains.

**Fig 3:**
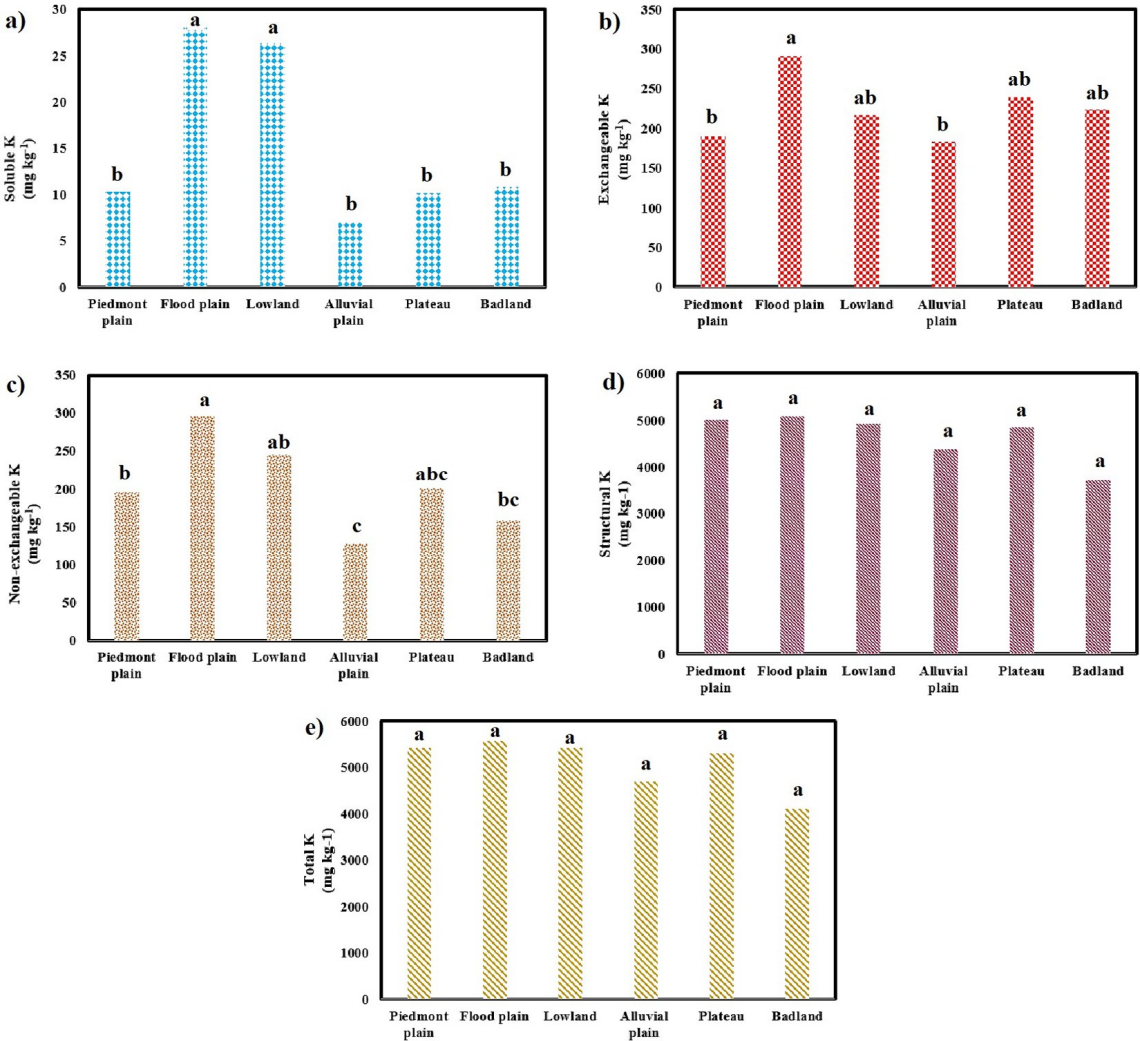
Comparing different forms of K in different studied physiographic units. a) soluble K, b) exchangeable K, c) non-exchangeable K, d) structural K, and e) total K content followed by different letters are significantly different at p<0.05 based on Duncan`s test.

The structural form of K ranged from 3193.40 to 6857.88 mg kg^-1^ with a mean value of 4755.50 mg kg^-1^. The structural form of K allocated 84.73 to 95.18% (with a mean value of 91.75%) of total K content to itself. The minimum and maximum amounts of structural K were found in the Bw horizon of profile no 11 and the Ap horizon of profile no 3, respectively. The trend observed for structural K form regarding the different physiographic units was as: badlands < alluvial plains < plateau < lowlands < piedmont plains < flood plains. The total K in the study area ranged from 3512.17 to 7571.06 mg kg^-1^ with a mean value of 5180.76 mg kg^-1^. The minimum and maximum amounts of total K were found in the Bw horizon of profile no. 11 and the Ap horizon of profile no 3, respectively. Regarding the different physiographic units, such a trend was found for total K content: badlands < alluvial plains < plateaus < lowlands < piedmont plains < flood plains. According to Fig 3A–3E, the mean values of all forms of K except structural K showed significant differences (*P* < 0.05) along different physiographic units.

### Correlation between different forms of K

Correlations between the selected physicochemical properties and different forms of K are shown in Table 4. The soluble K showed positive significant correlations with exchangeable (r = 0.732**), and non-exchangeable K (r = 0.762**), and total K content (r = 0.371*). In addition, the physicochemical properties such as silt (r = 0.511**) and gypsum (r = 0.475**), pH (r = 0.519**), EC (r = 0.723**), and SAR (r = 0.478**) showed significant positive correlations with soluble K forms; whereas, the percentage of sand had a significant negative correlation (r = -0.367*). Among all forms of soil K and physicochemical soil properties, only sand showed a significant negative correlation (r = -0.389*) with the exchangeable form of K. In addition, the exchangeable form of K showed positive significant correlations with soluble (r = 0.732**) and non-exchangeable K (r = 0.850**), silt (r = 0.593**), pH (r = 0.456**), EC (r = 0.617**), SAR (r = 0.500**) and gypsum (r = 0.384*). Non-exchangeable K showed a significant positive correlation with the total (r = 0.493**), soluble (r = 0.762**), and exchangeable K (r = 0.850**) and structural K (r = 0.357*). Silt (r = 0.527**), EC (r = 0.648**), and SAR (r = 0.577**) showed significant positive correlations with non-exchangeable K. In addition, significant positive correlations were observed between non-exchangeable K and gypsum (r = 0.372*) and pH (r = 0.347*), while a significant negative correlation (r = -0.357*) was observed between non-exchangeable K and sand. The structural K showed positive correlations with the total (r = 0.988**) and non-exchangeable (r = 0.357*) K contents, while no significant correlations were found with the studied physicochemical properties. Furthermore, total K content had no significant correlations with the selected soil physicochemical properties.

**Table 4:** Correlation between different forms of K and selected physiochemical properties.

	Exchangeable potassium	None-exchangeable potassium	Structural potassium	Total potassium	pH	EC	%CCE	CEC	%OC	%Clay	%Silt	%Sand	%Gypsum	SAR
**Soluble** **potassium**	0.732**	0.762**	0.256	0.371*	0.519**	0.723**	-0.099	0.003	0.104	0.026	0.511**	-0.367*	0.475**	0.478**
**Exchangeable potassium**	1	0.850**	0.109	0.254	0.456**	0.617**	-0.135	-0.024	0.335	-0.013	0.593**	-0.389*	0.384*	0.500**
**None-exchangeable potassium**	0.850**	1	0.357*	0.493**	0.347*	0.648**	-0.200	-0.013	0.226	-0.007	0.527**	-0.357*	0.372*	0.577**
**Structural potassium**	0.109	0.357*	1	0.988**	-0.018	0.027	0.014	0.196	-0.050	0.175	0.150	-0.193	0.154	-0.022
**Total** **potassium**	0.254	0.493**	0.988**	1	0.05	0.133	-0.015	0.183	-0.003	0.164	0.235	-0.245	0.210	0.069

EC: Electric Conductivity of saturated extract; CCE: Calcium carbonate equivalent; CEC: Cation exchange capacity; OC: Organic carbon; SAR: Sodium absorption ratio. * and **: Correlations are significant at the probability levels of 0.05 and 0.01, respectively.

## Discussion

### Soil classification of the study area

According to Table 1, the predominant soil texture in the study area was loam; besides other textures like sandy loam, silt loam, and silty clay loam were found. So, the upper geomorphic positions such as bad land (including hills) and plateaus had sandy loam texture due to the in-situ soil development and leaching rate. While lower geomorphic positions such as flood plains and lowlands had loam and silt loam textures mainly due to the leaching of finer particles (i.e. silt and clay) to the soil body from upper positions. The soil texture of middle geomorphic positions showed the intermediate state of both the other mentioned positions. The soils of the study area were relatively alkaline. The study area faced the salinity problem with the medium to high grade and the soils were also classified as calcareous soils based on the CCE values [40]. The percentage of gypsum in the study area was not enough to be classified as gypsiferous soil. In addition, some parts of the study area (i.e. profiles no 3, 4, 7, and 9) were affected by low sodium salts due to their SAR amounts. The organic carbon content was low due to the aridic conditions [41]. The CEC amounts were consistent with the percentage of clay and organic matter content. Topography affects soil erosion and deposition processes due to the dominant aridic soil moisture regime; besides, it modifies the distribution of soil horizons and properties [42,43]. Therefore, the current study area was not exempt from this issue, and soil development mostly affected by topography. Although the soils had been classified into Entisols and Aridisols orders, the effect of topography led to more variability in soil subgroups than the soil orders.

### Clay mineralogy

According to Table 2, the estimation of the relative abundance of clay fractions showed the presence of chlorite, illite, and kaolinite in almost all topsoil and subsoil horizons with moderate abundance. According to Khormali and Abtahi [44] statements, the abundance of chlorite in soils is largely related to its presence in the parent rock. In addition, this mineral is found naturally in sedimentary rocks and fertile soils derived from them. In the study area, the slight changes of chlorite with depths were resulted by neutral to alkali pH and its steady conditions in calcareous soils; and its presence had an inheritable origin. In addition, the pedogenic formation of kaolinite is impossible in soils with high pH. Thus, the presence of this mineral in the study area was due to its inheritable and detrital origins. The changes of illite with depths were also in agreement with its inherited origin in calcareous soil. The mixed interlayer minerals had a constant trend with increasing depths and were not present in large amounts. These minerals had an inherited origin. The present findings were in line with the observations of Owliaie et al. [45]. The presence of hormite clays (e.g. palygorskite) might be observed in the study area due to high EC. The neoformation of smectite (montmorillonite) occurs due to the presence of sufficient moisture resulted in release of K from illite, so the abundance of Mg and soil pH. Furthermore, transformation might be another main process for montmorillonite formation resulting from soil drainage. Thus, montmorillonite levels were related to parent materials and also had a constant trend with increasing with depths in the study area. These findings were in line with the observations of Shakeri and Abtahi [46].

### Distribution of different forms of K

According to Table 3, the observed differences in amounts of soluble K were mainly due to the plant uptake in upper horizons and the lack of sufficient moisture in aridic regions to transfer this K form readily downward with increasing depths. Similar observations were found by Li et al. [15]. In addition, another factor like the capability of organic matter in blocking specific and nonspecific K binding sites should be considered. This might be resulting in a reduced amount of K fixation and the sufficient amount of K remains in soluble form [47]. The soluble amounts of K showed significant differences in different physiographic units due to the slope impacts on runoff passing on the soil body resulting in nutrient leaching. Generally, the stable geomorphic positions are the places for accumulating leached materials from upper positions. This justifies our findings correctly.

The observed differences in the exchangeable form of K could be related to the presence of clay minerals containing K (e.g., illite) and their different weathering rate resulting from different soil development. However, other factors such as leaching rate, particle size distribution, cation exchange capacity, layer depth, pH, and the presence of phosphate compounds in the soil might affect these observations [48]. The observation of higher exchangeable K contents in the topsoil was mainly due to the application of K fertilizers, crop residue, higher organic carbon content, and higher biological activities of this layer [49]. Regarding different physiographic units, the differences might be observed due to the changes in amounts of illite and its weathering rate resulting from the combined impacts of topographic features (such as elevation, degree, and shape of slope) on soil development and eventually mineral distribution.

The non-exchangeable form of K is not accessible for plant uptake, but it takes part in the maintenance of exchangeable K levels [50]. Factors like leaching, clay type, and weathering of K-containing minerals in topsoil resulted in such differences in the study area. According to the findings of Rees et al. [16], the gradual release of the non-exchangeable K form also occurred when this form was fixed in the interlayer of 2:1 clay minerals. The non-exchangeable K had a systematic decreasing trend with increasing depth. This finding might be due to the accumulation of carbonatic and sulfatic minerals (e.g. calcite, dolomite, and gypsum) in soil depths. Although slight changes were observed in the mineralogy of different physiographic units, other factors such as particle size distribution and some chemical characteristics (e.g. the percentage of CaCO_3_ and CaSO_4_) might result in different weathering rates of K-bearing minerals controlling the gradual releasing of K from them [51].

The observed differences in the structural form of K were related to the increase in the weathering rate, soil development, and the instability of mica minerals and their decrement. In the study area, the less developed horizons (i.e. Bw) showed the minimum extracted structural K; while top soils showed the maximum extracted structural K due to being the interface of soil body and atmosphere where the climax of weathering occurred [52]. In the study area, this K form showed irregular trend with increasing with depth due to the presence of evaporitic minerals and the type of illuvial horizons identified in soil profile. In addition, different physiographic units produce different micro-climate resulting in nonuniform distributions of K-bearing minerals. The observation of non-significant differences between the amounts of structural K in different physiographic units might be due to the predominant effects of CaCO_3_ in soil development of the study area and the presence of illite, chlorite, and kaolinite with inherited origins. Since the major part of total K content comprised structural K, a non-significant difference was observed for total K content along different physiographic units. The current study confirmed the significance of the mean of K forms between physiographic units in southern Iran with Aridic moisture regime [14].

### Relationships between chemical forms of K and soil attributes

The correlations of soluble K showed that the finer sections of soils had a key role in supplying K for plants. In addition, these correlations showed that the increase in pH, EC, SAR, and gypsum led to an increase in the amounts of soluble K. These findings were logical due to the increasing amounts of these soil properties resulted in creating competition for binding to the exchangeable sites by the presence of cations such as Na^+^, Mg^2+^, and Ca^2+^ in soil solution and releasing K^+^ to the soil solution. According to Hayashi et al. [53], other factors such as K fertilizers, plant uptake, replacement by the other forms of K, and sampling time might affect the availability of the soluble K. Similar observations were found for exchangeable K, which might be explained by the same reasons due to the similar functions of these forms in supplying K for plants. This showed why these two forms of K are often known as one specific K form (available form) in scientific reports [54].

The correlations of non-exchangeable K showed that K-bearing minerals in the study area were presented in medium-sized fractions (silt) due to the low development of soils. In addition, soil properties such as pH, EC, SAR, and gypsum resulted in gradual releasing of the fixed K from these minerals and transferring to the exchangeable and soluble pools. These conditions might be recognized as initial conditions of illite decrement if the soil moisture and drainage were suitable [45]. The structural K had no significant correlations with soil properties due to having no enough effects on the distribution of this form in the study area. These findings might be occurred due to the inherited origins of K-bearing minerals such as illite. Similar findings with similar explanations were found for total K content due to the major parts of soil K in the study area comprised of structural K [45].

The correlations between different forms of K showed the dynamic equilibrium reactions between them in the studied soils, which was in line with previous studies [55]. According to Amoakwah and Frimpong [25], satisfaction of the nutritional demands of plants mostly depends on the soluble and exchangeable pools of K which are slowly depleted after prolonged periods of crop production and harvesting. If these K pools are depleted, plants can merely rely on the release of K from non-exchangeable pools to recharge the exchangeable and soluble pools.

## Conclusions

The present study showed that factors such as physicochemical properties, mineralogy, and topography (especially physiographic units) controlled the distribution of different forms of K in the study area. The physiographic units had significant effects on the soluble, exchangeable, and non-exchangeable forms of K due to controlling the run-off destiny on the soil body, leaching process, properties and distribution of soil minerals, and soil development. The total K content likewise structural K was not affected by any soil properties probably due to the inherited origin of soil minerals like illite. In arid regions like the study area, the physiographic unit functions as a microclimate that influences soil properties and development. This study suggests that farmers should select cultivation sites with a deeper understanding of land conditions, including not only slope and elevation but also microclimatic conditions, clay mineralogy, and soil development levels.

## Supporting information

S1 File Supplementary data. (XLS)
